# Dr. M. W. Wilson’s Clinical Reports

**Published:** 1842-06-18

**Authors:** M. W. Wilson

**Affiliations:** Resident Physician


### CLINICAL REPORTS.

### *Blockley Hospital—Service of W. W. Gerhard, M. D.*

Reported by M. W. Wilson, M. D., Resident Physician.

About 93 patients have been treated in four of the medical wards of this institution since April 18th, 1842, for a variety of complaints, most of them chronic. Some points of interest were, however, connected with them which we propose to notice briefly. Of phthisis pulmonalis there were 22 cases, 15 white males, four females, and three blacks.

One of the most interesting points observed in this class of diseases was an apparent reciprocal action between the lungs and kidneys. This was remarked in two cases. The first, H. M., entered the ward labouring under phthisis pulmonalis in its early stage. The disease gradually advanced after his entrance, until a crop of tubercular matter softened, and a cavity formed as evinced by the symptoms and physical signs. His treatment consisted of inhalations of the fumes of iodine and Lugol's solution of iodine internally. Under these prescriptions the patient appeared to be rapidly improving for several weeks, when a disease of the kidneys manifested itself; his urine became albuminous and rather scanty. General anasarca supervened, and that peculiar tint of the skin, a pale reddish colour, which so well characterises this disease—granular degeneration of the kidneys or morbus Brightii. As the disease of the kidney advanced, that of the lung declined, until the cough became insignificant. Treatment had but little or no effect on this disease, and the patient remained with no decided alterations in his general health for about two months, when he was attacked with ulceration of the bowels. Under this complication of diseases he gradually sank, and died seven months after his entrance in the ward—three months after the commencement of the last disease. An examination of his body, which would have been very interesting, was prohibited by his friends.

[*Remarks.*—From the symptoms which were observed in the last few days of life, it was plain that the immediate cause of death was the brain symptoms which gradually came on. As is usual in cases of kidney disease, the patient became dull, and gradually sank into coma. As far as the condition of his lungs could be ascertained by auscultation, the tuberculous deposit remained stationary. The influence of the iodine treatment seemed decided, so far as the pulmonary disease was concerned; but had not the decline of the lung symptoms a positive effect upon the complications which followed, and ought we not to ascribe these complications to the same cachexia, under a new form?—W. W. G.]

The next case, C. O., entered the ward labouring under diabetes in an advanced stage, and very much emaciated. He passed about twenty-seven pints in twenty-four hours at the time of his entrance, and took about the same quantity of fluid. The urine was limpid, and highly charged with saccharine matter. The disease came on eighteen months before his entrance, without assignable cause, and had not been modified by a variety of treatment which he had undergone.

He was put under the use of chalybeates, and his diet confined to animal food as exclusively as his stomach would bear. Under this management his general health improved, without materially diminishing the amount of his urine. In about eight weeks after his entrance an abscess became developed in the perineum, and he was transferred to the surgical ward. Here he was put on the use of wine and carbonate of ammonia, which enabled him to bear a more exclusive animal diet. In about a week his urine became reduced to about one-third of the amount he passed when he left the medical ward. The abscess was opened and continued to discharge until it finally closed, some weeks after, and the patient was transferred to the medical ward again for a cough, which was becoming troublesome.

It was found that phthisis pulmonalis was developing itself very fast. He passed about three quarts and a pint of urine in twenty-four hours. His urine continued to gradually diminish in quantity as the disease of his lungs advanced, until the amount of urine passed in twenty-four hours was reduced to a pint and a half. He was discharged from the hospital by his own request to remain among his friends, feeling that his case was near its termination.

Case III. *Phthisis following the healing of an old Ulcer.*—E. J., æt. 27, came to the medical from the surgical ward in February, 1842. He was treated in the surgical ward for an ulcer, which followed a slight wound on his shin two weeks before his entrance in the ward. An ulcer had followed a wound on his leg on two previous occasions, and lasted some three months before it healed. In June last he had an attack of disease, apparently gangrene of the lungs, lasting six weeks. After the healing of the ulcer on his leg, he was taken with chills and fever and night sweats. The chills came on with the regularity of intermittent fever. After attempting to regulate his digestive functions, he took quinine in full doses without relief, and was transferred to the medical ward. When first taken with these chills he had no cough, but at the time of his entrance into the medical wards he had some cough, and expectorated mucus like that of common bronchitis. The cough soon changed and become dry and short. Chills returned with regularity every morning, particularly on rising from bed. From the regularity this was supposed to depend upon the incipient but diffused tuberculation of the lungs. Respiration was enfeebled in the upper part of the left lung, and percussion was obscure. He was treated with emetics, which arrested the chills for a few days, but they returned when the emetics were discontinued. After this he was successively put on the use of quinine, cinchona, elixir vitriol, an infusion of the vegetable tonics, and the warm bath daily, but without the slightest benefit. Hectic fever, chills and night sweats continued, and with little alteration in his general symptoms, except gradual emaciation for three months after his entrance, when he had an attack of excessive hæmoptysis, preceded by a discharge of softened tubercular matter. He died about forty-eight hours after the hæmoptysis commenced. An examination of the body could not be obtained, but the case was plain enough ; rapid softening had followed extensive tuberculation, and a rupture of a bloodvessel resulted.

[*Remarks.*—These three cases of phthisis offer several points of interest. The reciprocal action of diabetes and phthisis is well known, and most cases of apparent cure of confirmed diabetes, in reality terminate in the worst form of phthisis. In the present instance the pectoral symptoms did not develop themselves until the discharge of urine had diminished ; was the treatment productive of this result by causing a favourable action on the flow of urine? Probably it was not, for the different modes of treatment adopted by various physicians for more than eighteen months, had been totally unsuccessful ; the change was, therefore, probably a natural one, independent of the action of remedies. There is another reason for taking this view of the subject. An abscess had formed in the perineum previously to the patient's transfer to a surgical ward. This abscess was one of the kind which, originating near the anus, are more frequent in phthisical patients than in any others, and seem to have a curative agency, or, at least, have some power of retarding the progress of the disorder ; when they heal up rapidly no benefit results ; on the contrary, the symptoms of phthisis become more urgent, and, as in the present case, sometimes assume one of the worst forms of phthisis, that is of general tuberculous infiltration.

In the other case, in which the symptoms of phthisis to a great extent ceased under treatment, and were succeeded by the albuminous disease of the kidneys, the action of remedies was much more apparent. The iodine acted gradually, and the cough and expectoration diminished little by little, but the general cachectic state of the patient did not improve ; still, his life was in all probability prolonged beyond the ordinary period of a tuberculous case.

In the third case, the tuberculous disease was equally grave, as in that of the diabetic patient, consisting of diffused infiltrated tubercles ; it was, however, slower in its progress. The unfortunate results of the cure of the ulcer are common enough, but do not always attract the attention of the surgeon, who is engrossed by the outward disease. The fever was extremely intense, and was in itself one of the worst symptoms of the disorder, for it will be found that cases of phthisis, in which the irritative fever of the early stages assumes the symptoms of a well defined hectic, with comparatively little local irritation, are always amongst the most intractable. In other words, the general disease predominates over the local lesion. Every conceivable remedy was tried to arrest the chills without permanent benefit ; by remaining in bed until a late hour the patient could generally diminish them, and sometimes escape a paroxysm,—but it was by no means a certain resource.

He was in the medical wards at two different periods. An issue was opened during the earlier part of the treatment, and the patient became well enough to leave the hospital for the benefit of a purer air; but on the increase of the symptoms, the pulmonary disease had evidently become independent of its canal.—W. W. G.]

Case IV. *Chronic Gastritis and commencing Cancer of the Stomach, complicating Phthisis Pulmonalis.*—This was a female of middle age and robust constitution, but whose habits were bad. When she entered the ward she vomited very frequently, and complained of pain in the epigastrium. She had a cough, not very troublesome, however, and expectoration of tuberculous matter. Various means were taken to avert the vomiting, but they were ineffectual. Near the conclusion of the disease she vomited almost incessantly. She died about five weeks after her entrance.

On examination of the body, the lungs were found in an advanced stage of phthisis. A cavity existed in the upper lobe of the right lung, and tubercular deposits throughout both lungs. The mucous membrane of the stomach was softened, and of a dark slate colour. At the pylorus the orifice was almost entirely closed by a thickening and induration of the tissue, apparently the commencement of schirrus.

The other cases of phthisis presented no points of interest. Their history is like that of the majority of this class of patients whom we meet with in the wards of this institution. Many were drunkards, irregular in their habits, and exposed to every vicissitude of weather and improper food, until their constitution becomes broken down, its energies wasted, and in many, from a specific cause, the system is vitiated, so that they enter and die after a few months' suffering.

*Pneumonia*, during this season, has been mostly complicated with other diseases. But two cases came in these wards during the last seven weeks. They were blacks, and the disease exhibited the adynamic form, requiring stimulants from the commencement of the attack. Both cases convalesced under the use of wine and of stimulating expectorants, composed of muriate of ammonia and syrup of senega.

Of *Bronchitis* three cases in all entered,—two blacks and one white man. They were simple cases and void of interest.

*Laryngitis.*—One case of severe laryngitis and bronchitis terminated fatally. The patient had originally entered the ward in the commencement of the winter, was treated by repeated leeches to the neighbourhood of the larynx, and by the application of caustic (nitrate of silver,) to the pharynx as a revulsive. Under this treatment the patient convalesced, the cough ceased, and he regained his flesh, but remained aphonic. He was discharged, but remained in the ward as an assistant nurse for two or three months. The ulcerations of the larynx, it was believed, had destroyed the vocal cords, and had left cicatrices of the larynx. The patient afterwards quitted the house, and after exposure was again attacked with inflammation of the larynx and of the bronchial tubes. After free cupping and two applications of leeches to the throat, with antimonials, the patient was partially relieved, the dyspnœa diminished, and the wheezing, which not only produced an intense sibilant rhoncus, but could be heard at a distance of some feet from the patient, was much lessened. The application of the nitrate of silver was then made to the pharynx, and a blister was applied over the anterior part of the thorax. The application of the caustic, instead of producing relief, gave rise to severe spasmodic coughing, so much as to render it possible that a drop of the liquid had fallen into the larynx from some spasmodic contraction of the parts during inspiration. The patient finally died of suffocation. The operation of tracheotomy was not attempted, because the inflammation was obviously not limited to the larynx, but involved the trachea and bronchi.

No examination was made, owing to some accidental circumstances, but there was every reason to believe, from the extent of the inflammation, the violence of the symptoms, and the glairy matter discharged from the larynx, that it was a case of pseudo-membranous inflammation. Upon the recurrence of the severe symptoms mercurials were given, but not for a sufficient time to produce any positive effect.

*Endocarditis.*—Of this inflammation four cases entered the wards, one woman and three blacks. The first was a mild case, and yielded readily to treatment. Among the blacks the disease assumed a more grave character from its extensive complications. Some of the interesting points of these cases will be noticed. For the first of these cases the reader is referred to the 22d No. of the Examiner.

The next case, E. J., a robust negro, entered the ward on the 21st of May. He was taken suddenly ill on the evening of the 16th with cephalalgia and fever, followed next day by pain in his knee joints, thirst, loss of appetite, and dyspnœa. He had no chills; no treatment except sinapisms to the temples, which vesicated, and were discharging when he entered the ward.

May 21st. *Present state.*—Decubitus slightly elevated; expression anxious; dyspnœa; nostrils dilated; eyes suffused; conjunctivæ injected and slightly jaundiced; tongue white in the centre, and red at tip and edges; skin dry and harsh; pulse 128, irregular, and almost imperceptible; respiration 50, chiefly abdominal; extreme cutaneous sensitiveness over abdomen; no pain in chest or abdomen; slight cough; no expectoration. The physical signs indicated pleuritis, with effusion on the right side, and the first stage of pneumonia on the left, with endocarditis. He was treated with local depletion and laxatives, and stimulating pediluvia night and morning.

No change of importance occurred in his symptoms until the third day after his entrance, when his pulse became more developed; dyspnœa increased, and he had slight subsultus tendinum. He could be bled to fourteen ounces, which made a decided impression on his pulse, and was ordered half a grain of calomel every four hours. In the evening the patient was much relieved; he had no subsultus; respiration 36; dyspnœa slight; pulse 112;

Local depletion was still made use of, with the pediluvia and calomel. From this time the patient improved gradually. At the end of five days all remedies were discontinued. He is now quite well.

Case 3d was a coloured man of small stature, æt. 21. He entered the ward on the 23d of May. He was taken ill on the 16th with violent cephalalgia, followed next day by pain in his joints. He became dull and sleepy, and he says that part of the time he was delirious. Cough commenced two days previous to his entrance, with slight expectoration. He has taken no medicine but a dose of oil.

When he entered the ward he had expression of dyspnœa; (nostrils dilated;) conjunctiva brightly injected; tongue white; decubitus natural; respiration hurried, mostly abdominal; slight cough; expectoration viscid and rust coloured; pulse quick, irregular, and almost imperceptible. The physical signs indicated extensive pneumonia of the right lung, with bronchitis; and also endo-pericarditis, with effusion in the pericardium.

His treatment was local depletion from the chest, stimulating pediluvia night and morning, and half an ounce of the liquor ammoniæ acetatis every two hours. The next day delirium commenced, with stupor; no other alterations in the symptoms. For this he was cupped and blistered to the nucha, and had ice applied to his head. On the following day his prostration was extreme. He was directed a clyster of half an ounce of turpentine and three ounces of mucilage three times a day, but it produced little or no benefit. He died about five days after his entrance in the ward.

On examination, after death, the arachnoid membrane was found extensively inflamed over both hemispheres of the cerebrum, and the substance of the cerebrum unusually injected; no fluid in the ventricles. The pericardium contained about half a pint of reddish serum, and the internal membrane of the heart, in both ventricles, was intensely reddened with deposition of lymph among the columnæ carneæ. On the left side the inflammation extended up the aorta for some distance. The valves were flexible. The right lung was granulated throughout the middle and lower lobes. Pleura was very extensively adherent. The left lung crepitated but feebly on pressure—very much congested. The lining membrane of bronchia minutely injected, and of a bright red colour.

Endocarditis, it will be seen from these cases, is a very common disorder, and not a grave one, unless complicated with other inflammations, especially pneumonia. In the latter case it forms merely one of a series of inflammatory phenomena, the blood participating in the disorder as much as the solid tissues. But when endocarditis is simple, the common antiphlogistic treatment quickly relieves it.

